# Using C. elegans to Find Genes, Mechanisms, and Drugs to Slow Cognitive Decline

**DOI:** 10.1093/geroni/igaa057.2627

**Published:** 2020-12-16

**Authors:** Coleen Murphy

**Affiliations:** Princeton University, Princeton, New Jersey, United States

## Abstract

Cognitive decline is one of the most important losses of human quality of life with age. To find new treatments that might slow these declines, it is useful to have short-lived, rapidly aging models in which to identify possible treatments. We found that C. elegans also lose their ability to learn and remember with age, along with other neuronal functions (axon regeneration, motility, etc.), and these declines are staved off by reduction in the insulin signaling pathway, which also extends lifespan. Both the mechanisms that regulate neuronal functions and the mechanisms that slow decline are well conserved evolutionarily, suggesting that we can use worms to identify new genes and compounds that might slow decline. Additionally, we have developed new C. elegans models of Parkinson’s and Alzheimer’s diseases, and through genetic, genomic, and metabolic studies we have found new approaches that may slow these declines.

